# Complex coacervation of soy protein isolate‐limited enzymatic hydrolysates and sodium alginate: Formation mechanism and its application

**DOI:** 10.1002/fsn3.3009

**Published:** 2022-08-07

**Authors:** Min Xu, Jiayi Li, Ying Wang, Jiamin Liu, Ping Liu, Qin Wang, Zhenming Che

**Affiliations:** ^1^ School of Food and Bioengineering Xihua University Chengdu China; ^2^ Department of Nutrition & Food science University of Maryland College Park Maryland USA

**Keywords:** enzymatic hydrolysis, soybean protein isolate, sodium alginate, complex coacervation

## Abstract

The complex coacervation of soybean protein isolate and polysaccharide has been widely applied for preparing biopolymer materials like microcapsule. In this study, hydrolytic soy protein isolate (HSPI) was prepared by mild hydrolysis of soy protein isolate (SPI) with fungal protease 400 (F400). The degree of hydrolysis (DH) for the enzymatic products was controlled at 1%–5%. Emulsification, oxidation resistance, and thermal stability were used to evaluate the performances of HSPI with different DH. The results showed that the HSPI with the hydrolysis degree of 2% had the optimal property. Subsequently, the complex polymer of HSPI/SA was prepared by the coalescence reaction of HSPI and sodium alginate (SA). The turbidity curves manifested the optimal complex coacervation occurred at the ratio of 7:1 (HSPI:SA). Fourier transform infrared spectroscopy (FTIR) presented that the reaction involved electrostatic interactions between ‐NH_3_^+^ in HSPI and ‐COO^−^ in SA. Isothermal titration calorimetry experiments indicated that the complex coacervation reactions of HSPI and SA arose spontaneously. The microencapsulation by complex coacervation of HSPI and SA was further produced for embedding sweet orange oil. The thermogravimetric analysis (TGA) result revealed that the microencapsulation system of HSPI/SA had a better heat resistance than that using the SPI/SA complex polymer.

## Graphical abstract

The fungal protease 400 was applied to mildly hydrolyze soybean protein isolate(SPI) for improving its solubility and emulsifying properties. Afterwards, the hydrolytic soybean protein isolate (HSPI)/sodium alginate (SA) polymer was prepared by complex coacervation reaction and the formation mechanism of the reaction was investigated. The HSPI/SA complex achieved better heat stability than SPI/SA complex when they were applied as microcapsule wall material for the embedding of sweet orange oil.

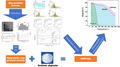

## 1 INTRODUCTION

The protein and polysaccharide are widely used biopolymers in food systems. The complex coacervate of protein and polysaccharide often provides superior physicochemical properties than the single macromolecule in food gel system (Nishinari et al., 2000; Yang et al., 2021), emulsion system (Ge et al., 2021; Ribeiro et al., 2021; Wong et al., 2021), and drug delivery system (Bealer et al., 2020; Zhang, Hao, et al., 2021b). Complex coacervation is driven by the interaction of two oppositely charged colloids (De Kruif et al., 2004). The amino groups of proteins can bind to the negatively charged free carboxyl groups in polysaccharides through electrostatic attraction and hydrogen bonding interactions (Gentile, 2020).

The formation of protein–polysaccharide complexes could bring about better functional properties of solubility, emulsification, and thermal stability than individual polymer system (Xu et al., 2017). For example, the solubility of pea protein isolate (PPI)–maltodextrin conjugate was higher than that of PPI itself (Kutzli et al., 2020). As (Jayakumar et al., 2014) reported, the denaturation temperature and stability of collagen were altered by forming collagen–pectin complex. Soybean protein isolate (SPI) is produced from soybean meal. The abundance of essential amino acids in SPI makes it the rare vegetable protein that can replace animal proteins (Ma et al.,  2015). In order to obtain better properties, more and more researches concentrated on the construction of the polymerization system of SPI and polysaccharides, such as the SPI/chitosan (Wu et al., 2018), SPI/gelatin (Liao et al., 2009), and SPI/sodium dodecyl sulfate complex systems (Xiaoli et al., 2009).

The hydrolysate modification of SPI is expected to expose more charged amino acid side chains, thus could provide more interactions with polysaccharides than SPI (Tavernier et al., 2017). Currently the modification of soybean isolate, mainly including physical, chemical, and bioenzymatic methods, attracts wide attention from researchers (Bu et al., 2022; Zhang et al., 2015; Zhang, Cheng, et al., 2021a). The physical methods have no significant improvement on the functional properties of SPI such as the antibacterial and antioxidant properties. Meanwhile the chemical modification methods may produce toxic derivatives. Bioenzyme is an effective modification method with mild treatment conditions (Sun‐Waterhouse et al., 2014). Furthermore, the bioenzymatic modification of SPI has been found to reduce molecular weight, as well as improve its solubility and emulsifying activity (Lopes‐da‐Silva & Monteiro, 2019; Sun, 2011), which provides a feasible method for obtaining a more stable system of complex polymer. However, few studies have been reported on the complex coacervation of the modified SPI with polysaccharides.

Natural polysaccharides are diverse and widely available polymers that consist of aldoses or ketoses of the same or different monosaccharides linked by α‐ or β‐glycosidic bonds (John & Zhong, 2019). Sodium alginate (SA) is a linear polysaccharide obtained from seaweed (Lacerda et al., 2014). The electrostatic attraction between the carboxyl group of SA and the amino group of HSPI might be conducive to the construction of complex polymeric system.

Hence, in this study, the protease F400 was used for the limited hydrolysis of SPI and the corresponding hydrolysates (HSPI) were obtained. Afterward, the HSPI was aggregated with SA for the preparation of HSPI/SA complex coacervate microcapsule. The main purpose of this study is to investigate the formation mechanism of the HSPI/SA complex system. Meanwhile the application of HSPI/SA complex as microcapsule for embedding sweet orange oil was conducted.

## 2 MATERIALS AND METHODS

### 2.1 Materials

Commercial SPI was provided by Shandong Wonderful Industrial. The protein content of SPI was 91.84% ± 2% (w/w on dry basis) based on Kjeldahl method. SA of analytic grade was purchased from Solarbio Science & Technology Co., Ltd. The fungal protease 400 was supplied by Enzyme Development Corporation. All aqueous solutions were prepared with deionized water (Milli‐Q water).

### 2.2 HSPI preparation and characterization

#### 2.2.1 Enzymatic hydrolysis of SPI

The commercial SPI (5.0 g) was dispersed in 100 ml of deionized water and stirred for 1 h at room temperature to ensure complete rehydration. The pH value of the suspension was adjusted to 7.0 with 0.5 M NaOH and then F400 was added according to the enzyme to substrate ratio (E/S) of 2 g/100 g. Subsequently, the enzymatic hydrolysis was carried out at 55°C with agitation and the degree of hydrolysis (DH) was determined, until the DH of 1%, 2%, 3%, 4%, and 5% were obtained. The reactions were terminated by heating in boiling water of 100°C for 10 min to inactivate the enzyme. The cooled hydrolysates were centrifuged at 3500 rpm for 15 min to remove impurities. The supernatants were freeze‐dried and stored at −20°C for further application.

#### 2.2.2 The degree of hydrolysis

DH was defined as the ratio of the peptide bonds broken number to the total number of bonds per unit weight (h_tot_), and was determined using the pH‐stat method and given by the following calculation equation (Zhao et al., 2012):

$$\mathrm{DH}=\frac{B\times N_{b}}{\alpha \times M_{p}\times h_{\mathrm{tot}}}\times 100\%\;\;\left(1\right)$$

where *B* is the consumption of NaOH, *N*_b_ is normality of the base solution, *α* is average degree of dissociation of the α‐NH_2_ groups, *M*_p_ is the mass of protein, *h* is the hydrolysis equivalents in meqv/g protein, and *h*_tot_ is total number of peptide bonds in the protein substrate (7.8 meqv/g soy protein).

#### 2.2.3 Emulsifying measurement

The emulsifying activity index (EAI) and the emulsion stability index (ESI) were determined based on the method provided by Lopes‐da‐Silva and Monteiro (2019). The emulsion was prepared by mixing 75 ml of 0.1 wt% HSPI dispersion in 0.05 mol/L Tris‐HCl buffer with 25 ml of soybean oil. Next, the emulsion was homogenized in a T18 Ultra‐turrax homogenizer (IKA‐Werke, Germany) for 1 min at 10,000 rpm. Emulsion aliquots (50 μl) were taken immediately (*t*_0_ = 0 min) and after 10 min (*t*_10_ = 10 min), and then the aliquots were diluted in 0.1 wt% sodium dodecyl sulfate (SDS) solution with the volume ratio of 1:100 (v/v). Subsequently, their absorbance values were read at 500 nm using a SpectraMax i3x Microplate Reader (Molecular Devices). The EAI and the ESI were calculated by the following equations:

$$\begin{array}{c} \mathrm{EAI}\left(\frac{m^{2}}{g}\right)=\frac{2\times 2.303}{C\times \left(1-\varphi \right)\times 10^{4}}\times A0\times \text{dilution}\;\;\left(2\right) \end{array}$$

$$\begin{array}{c} \mathrm{ESI}\left(\min\right)=\frac{A_{0}\times t}{A_{0}-A_{t}}\;\;\left(3\right) \end{array}$$

in which *A*_0_ and *A*_t_ are the absorbance at 500 nm of the diluted emulsions at *t*_0_ and *t*_10_ (*t* is 10 min), *C* represents the HSPI concentration (g/ml) before emulsification, and φ is the oil volume fraction (v/v) of the emulsion.

#### 2.2.4 Determination of heat stability

HSPIs with different degrees of hydrolysis were dissolved in 0.01 M phosphate buffer of pH 7 to prepare a 5 mg/ml solution. Then, ultrasonic processing was carried out at room temperature for 15 min and stirred till fully dissolved. Protein solution (90 ml) and corn oil (10 ml) were emulsified at a speed of 12,000 rpm for 3 min to obtain a protein emulsion. The protein emulsions were severally bathed at 30, 50, 70, and 90°C for 30 min. Then, the emulsion samples were cooled down to room temperature and kept for 24 h. Particle size distribution analysis was carried out using a dynamic light scattering (DLS) instrument (ZS90, Malvern Panalytical Ltd.). The ζ potential for each sample was measured by the Malvern Zetasizer Nano ZS90 (Xu et al., 2016).

#### 2.2.5 Molecular weight (MW) determination

The sodium dodecyl sulphate–polyacrylamide gel electrophoresis (SDS‐PAGE) was performed to obtain the MW information of HSPI based on the method described previously (Laemmli, 1970). HSPIs were dissolved in 0.0625 M Tris‐HCl buffer solution of pH 6.8 to prepare a 10‐mg/ml dispersion agent, in which 2% (w/v) SDS, 5% (v/v) mercaptoethanol, 25% (v/v) glycerol, and 0.01% (w/v) bromophenol blue were added. The mixtures were heated at 95°C for 5 min, and then were centrifuged at 12,000 *g* for 30 min at 20°C. The supernatants were reserved for further application. The sample load was 10 μl. Gel electrophoresis was carried out under constant current, the current was 10 mA in the concentrated gel and increased to 20 mA after the separation gel entered. The concentration of separated gel is 12% and the concentration of the concentrated gel is 5%. After electrophoresis, the gel was taken out. Coomassie stain (0.25%) was used for 1 h and then the sample was decolorized using the solution containing 7% acetic acid and 10% methanol. The MW range of standard protein sample was 14.4 to 120 kDa.

#### 2.2.6 Inhibiting activity of lipid oxidation

Anti‐lipid peroxidation is an important indicator of antioxidant capacity, which was determined according to the method provided by Zhang et al. (2014). Lecithin was dissolved in 0.01 mol/L phosphate buffer of pH 7.4 to prepare a 10 mg/ml solution (labeled as LLS). Briefly, 15 g of trichloroacetic acid (TCA), 0.37 g of thiobarbituric acid (TBA), and 2 ml of concentrated hydrochloric acid were added in distilled water to prepare a 100 ml solution (labeled as TCA/TBA/HCl). LLS (1 ml), 400 μmol/L FeCl_3_ (1 ml), and sample solution (1 ml) were orderly added into the test tube and then placed in a 37°C water bath for about 60 min (Zhang et al., 2014). Subsequently, a boiling water bath for 15 min was carried out after adding 2 ml TCA/TBA/HCl, then removed in ice water to cool down. The pink solution was centrifuged at 8355 g for 10 min. Afterward the supernatant was taken out for the absorbance measurement at 532 nm with a UV‐2450 spectrophotometer (Shimadzu). In the blank sample, the supernatant was replaced by distilled water. Antioxidant capacity was calculated according to the following equation:

$$\begin{array}{c} \text{Inhibiting activity of lipid oxidation}\left(\%\right)=\frac{A_{C}-A_{S}}{A_{C}}\times 100\;\;\left(4\right) \end{array}$$

in which $A_{C}$ indicated the absorbance value of blank sample, correspondingly, $A_{S}$ was the absorbance value of supernatant sample.

### 2.3 Characterization of the HSPI/SA

#### 2.3.1 ζ‐potential measurements

The reaction solution with 0.01% (w/v) concentration was prepared by dissolving HSPI in deionized water. A 0.01% (w/v) SA solution was prepared by the same method. Acetic acid solution was used to adjust the pH value for preparing the samples with the pH gradient varied in the range of 2.5–6.0. The ζ‐potential value of each sample was measured by the Malvern Zetasizer Nano ZS90 (Malvern Panalytical Ltd.).

#### 2.3.2 Turbidity measurements

The HSPI and SA solutions (0.01%, w/v) as prepared before were mixed at different ratios (5:1, 7:1, 9:1, w/w) to a total concentration of 0.1%. The initial pH of sample was adjusted to 6.0 by adding 0.1 M NaOH. Afterward the pH of the solution was slowly regulated to 2 with the addition of acetic acid. And the absorbance values of the solutions with different pH during the regulation were measured at 600 nm using a UV‐2450 spectrophotometer (Shimadzu) (Klemmer et al., 2012).

#### 2.3.3 Fourier transform infrared spectroscopy (FTIR)

FTIR measurement was carried out using a Nicolet 6700 spectrophotometer (Thermo Fisher Scientific). Appropriate amount of DH 2% HSPI, SA powder, and freeze‐dried HSPI/SA complex samples (the HSPI/SA was prepared by mixing HSPI and SA at the ratio of 7:1 to a total concentration of 0.1% and the pH was adjusted to 3.5) were severally crushed with KBr to produce a transparent sheet for analysis. Blank KBr was taken as control. Then, the spectra in the range of 500–4000 cm^−1^ with an average of 32 scans at the resolution of 4 cm^−1^ were recorded.

#### 2.3.4 Isothermal titration calorimetry (ITC)

ITC measurements were performed with a VP‐ITC calorimeter (Malvern Panalytical Instruments Corp.). The HSPI and SA at pH 3.5 were fully dialyzed for 24 h with deionized water and the dialysis water collected as the diluted solution. After that, the concentration of HSPI was analyzed by the Kjeldahl method (Weesepoel et al., 2019), while the SA concentration was measured with the phenol–sulfuric acid method (Zhang et al., 2020). The HSPI and SA solutions were adjusted to 0.1% (w/v) using the dialysis water. All solutions were degassed at 20°C for 5 min before analysis. The HSPI solution was added to a measuring cell of 1400 μl at 25°C, and the SA was taken into a specific syringe of 300 μl. The speed of measuring cell was 260 rpm. Each titration volume was 10 μl and 30 times conducted, and the titration interval was 240 s. For the same reaction system, the temperatures of 25°C, 35°C, and 45°C were set for analysis.

### 2.4 Preparation of microcapsules and property analysis

#### 2.4.1 The preparation of microcapsules

The same mass of HSPI or SPI were mixed with SA in the ratio of 7:1 and dissolved in deionized water to a total mass concentration of 10 g/L. The pH was slowly adjusted to a specific value with acetic acid solution at a stirring rate of 500 rpm for 30 min to obtain the corresponding complex solution, which was freeze‐dried in a further step to produce the solid powders of SPI‐SA and HSPI/SA.

Sweet orange oil (1 ml) was severally added to the prepared SPI/SA and HSPI/SA mixture of 100 ml, and dispersed at a high speed for 3 min at 10,000 rpm. Furthermore, the HSPI/SA and SPI/SA solid powder microcapsules were obtained by freeze‐drying.

#### 2.4.2 Thermogravimetric analysis of microcapsules

About 5 mg of sweet orange oil and freeze‐dried sweet orange oil microcapsules were taken separately and their heat release curves were measured using a DTG‐60 simultaneous thermal analyzer (Shimadzu) under the following conditions: initial temperature of 25°C, ramp up to 400°C at a rate of 20°C/min, and nitrogen at a flow rate of 30 ml/min.

### 2.5 Statistical analysis

The measurements were carried out in triplicates for samples, and values were expressed as mean values ± SD. Data were analyzed by SPSS Statistics 16.0 (International Business Machines Corporation).

## 3 RESULTS AND DISCUSSION

### 3.1 The results provided by SDS‐PAGE

**FIGURE 1:**
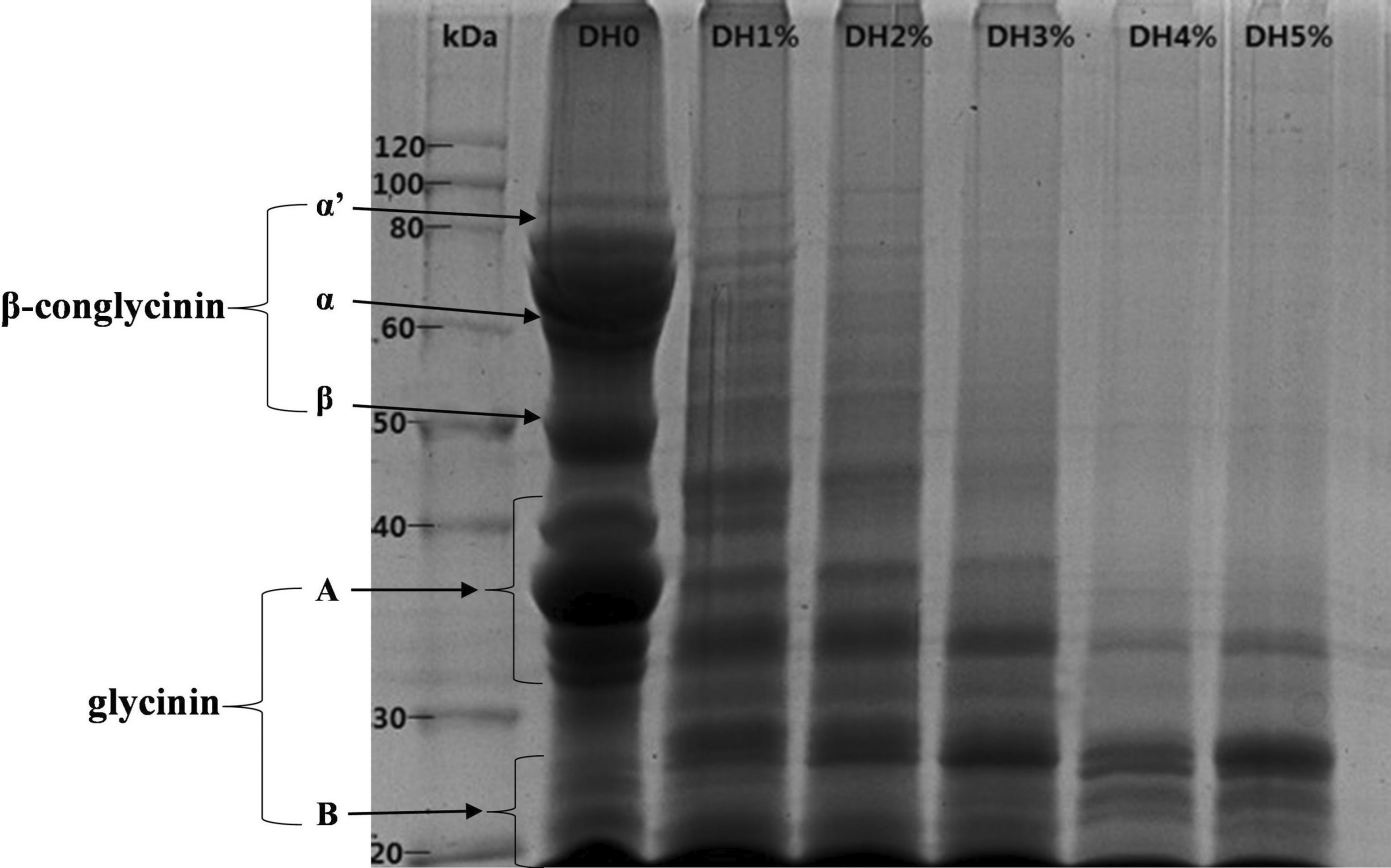
SDS‐PAGE profile of HSPIs obtained with different degrees of enzymatic hydrolysis. α’, α, and β indicate subunits of β‐conglycinin (7S). A and B indicate acidic and basic polypeptides of glycinin (11S), respectively

The complex and subunits of protein could be quantified using SDS‐PAGE. Figure 1 presented the SDS‐PAGE profiles of SPI and its hydrolysis products. It was found that the MW of the proteins decreased with the increase of enzymatic hydrolysis degree. In particular, the 7S protein (β‐conglycinin) was mainly composed of α (66 kDa), α’ (86 kDa), and β (51 kDa) subunits, while acidic (34–43 kDa) and basic (17–26 kDa) subunits were mainly present in the 11S protein (glycinin) (Liu et al., 2007). As presented in Figure 1, SPI illustrated a wide variety of polypeptide subunits of molecular weight ranging from 20 to 80 kDa, indicating that both 7S and 11S are enriched in the original protein. As the DH of the SPI increased from 1% to 5% (lanes 3–7), the molecular weight bands between 30 and 80 kDa decreased significantly, while the intensity of the protein bands that were lower than the 28 kDa increased. The results obtained above demonstrated that F400 preferentially hydrolyzes the 7S protein and the basic subunit of the 11S protein was mainly retained.

### 3.2 Emulsifying properties of SPHs

**FIGURE 2:**
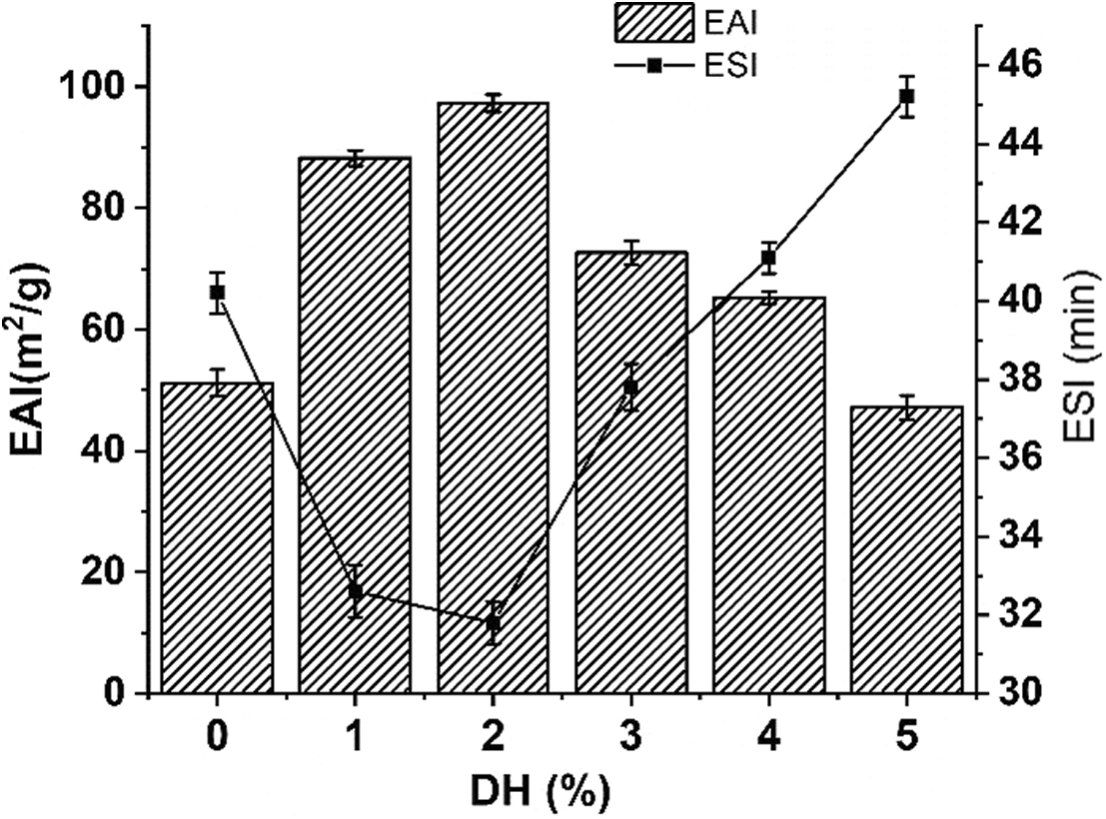
Emulsifying activity index and ESI of HSPI prepared using different degrees of enzymatic hydrolysis

Emulsion activity index (EAI) and emulsion stability index (ESI) were applied as reliable indicators for characterizing protein emulsifiers. Figure 2 illustrated the EAI and ESI of the original soy protein isolates and its enzymatic digestion products with F400 at different DHs. As presented in Figure 2, the EAI increased markedly from 51.15 m^2^/g to 97.28 m^2^/g with the increase of DH, and the highest EAI value could be obtained at the DH of 2%. This might be ascribed that molecular flexibility of polypeptides was increased and hydrophobic areas were exposed due to hydrolysis (Ghribi et al., 2015). However, the further increasing of DH from 2% to 5% led to the decrease of EAI. This is in accordance with the previous results (Wasswa et al., 2007), presenting that a low DH of protein is sufficient to improve EAI. An inverse relationship between ESI and EAI values was found from Figure 2. When the DH was 2%, the ESI achieved its lowest value and the EAI reached its maximum. The result suggested that the enzymatic hydrolysis improved the emulsifying ability of SPI at the DH of 2%.

### 3.3 Analysis of heat stability

**FIGURE 3:**
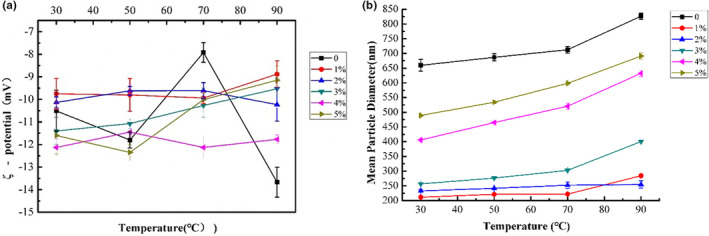
Analysis of heat stability with different enzymatic hydrolysis degrees (SPI, DH1%, DH2%, DH3%, DH4%, and DH5%): (a) ζ potential, (b) mean particle diameter

Thermal stability of the protein hydrolysates could be reflected by the mean particle diameter and ζ‐potential index. Figure 3a,b severally illustrated the mean particle diameter and ζ potential of samples with different enzymatic hydrolysis degree. As found in Figure 3a, the ζ potential of SPI emulsion varied strongly with the increase of temperature. The protein hydrolysates with 1% and 2% DH were relatively stable to heat processing. Meanwhile, the emulsions with higher DHs presented much more significant changes in ζ‐potential values with the increase of temperature. The result is consistent with the finding of Zang et al. (2019).

As observed from Figure 3b, the mean particle diameter of the HSPI increased with the increase of temperature. It might owe to the exposure of the hydrophobic groups resulting in the increasing of electrostatic attraction and the formation of disulfide bonds of proteins, which promoted the aggregations among particles (Charoen et al., 2011). Generally, it could be found from Figure 3b that the average particle size of the HSPI with 2% DH was relatively stable with the change of temperature. In addition, the HSPI with higher DHs showed a relatively larger particle size distribution. This might be because of the exposure of hydrophobic groups and the formation of insoluble aggregates as the DH increased (Kuipers et al., 2007; Kuipers & Gruppen, 2008). Similar results could be observed in the researches of Shen et al. (2020) and Zang et al. (2019).

### 3.4 Inhibiting activity of lipid oxidation

**FIGURE 4:**
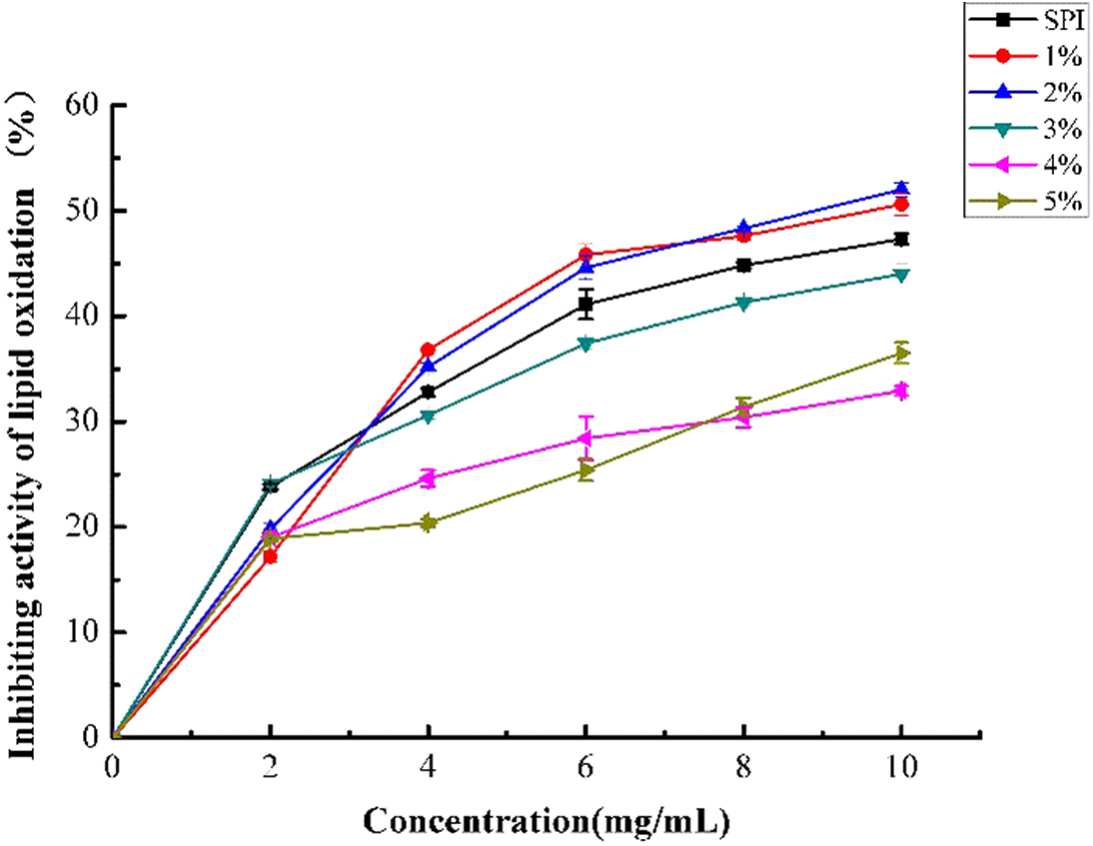
Inhibiting activity of lipid oxidation of HSPI under different enzymatic hydrolysis degrees

The HSPI with a high resistance to lipid oxidation is particularly important for the protection of the hydrophobic core. In free radical reaction, the produced hydroperoxide was unstable and decomposed promptly into shorter chain hydrocarbons. The malondialdehyde produced from the oxidation reaction could be determined with thiobarbituric acid reactive substances for evaluating lipid peroxidation inhibition capacity. As shown in Figure 4, the lipid antioxidant capacity increased with the increase of concentrations (0–10 mg/ml). The enzymatic hydrolysis disrupted the intrinsic structure of soy protein, which led to the exposure of active amino acid residues that could react with oxidants (Ren et al., 2021). Therefore, enzymatic hydrolysis has a positive influence in improving the resistance to oxidation. As illustrated in Figure 4, the hydrolysis products from DH3, DH4, and DH5 decreased the inhibition of lipid peroxidation when compared with SPI. This might be explained that the excessive enzymatic digestion destroyed the active sites in peptides that could react with fatty acids, thereby reducing their inhibitory effect on lipid peroxidation. In contrast, DH1 and DH2 showed significant improvement in antioxidant capacity, suggesting that mild SPI hydrolysis improved its ability to resist lipid oxidation.

Overall, the SPI that was hydrolyzed with F400 at 2% DH had favorable properties of emulsibility, lipid peroxidation inhibition, and thermal stability. Therefore, the HSPI with 2% DH was chosen in the following research for preparing polymer complexes with a polysaccharide.

### 3.5 Effect of pH on ζ potential and turbidity of the HSPI/SA

**FIGURE 5:**
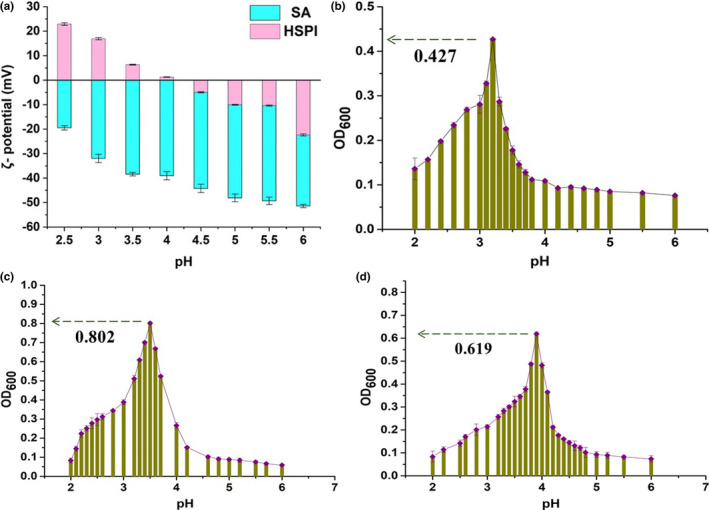
ζ potential and turbidity changes for HSPI and SA with different pH:(a) ζ‐potential changes for HSPI and SA, (b) turbidity changes with the HSPI:SA of 5:1, (c) turbidity changes with the HSPI:SA of 7:1, (d) turbidity changes with the HSPI:SA of 9:1

Figure 5a showed the ζ‐potential changes of the HSPI and SA as the pH varied. It was noticed that the ζ potential of HSPI increased from 0 mV to +23 mV as the pH value decreased from 4.2 to 2.5. The electrostatic charge was zero at the pH of 4.2, indicating the pH of HSPI was around 4.2. Therefore, the HSPI carried positive charges when the pH was below 4.2. On the other hand, it could be found that SA carried negative charges in the pH range of 2.5–6.0. The results of ζ potential implied the electrostatic driving force for complex coacervate reaction between the positively charged HSPI and the negatively charged SA in the pH range of 2.5–4.2.

Turbidity is a primary indicator for the research of complex coacervation, and the mixture with the highest turbidity is generally regarded as the optimal condition of complex coacervates (Alberti et al., 2019). The turbidity curves of the HSPI and SA complex coagulation products at different mass ratios were obtained as a function of pH, and the optimum HSPI/SA ratio was selected for the subsequent study. Figure 5(b‐d) showed the variation of turbidity at three different mass ratios of HSPI/SA (5:1, 7:1, and 9:1). It was seen from Figure 5(b‐d) that the turbidity reaction started at pH 6.0. The mixture was homogeneous and transparent due to the electrostatic repulsion of HSPI and SA. As the pH decreased, the optical density value (OD_600_) increased since weakly electrostatic attraction between the positively charged protein molecules and the negatively charged SA. That was the stage of soluble polymer formation and the corresponding pH value was defined as pHc (Mekhloufi et al., 2005). As shown in Figure 5(b‐d), the OD_600_ rose rapidly to 0.427, 0.802, and 0.619 throughout the pH range of 4.5–3 with the HSPI/SA ratio of 5:1, 7:1, and 9:1, respectively. The result indicated that the insoluble coacervates began to form around pH 4.5, at which the opaque solution of milky white formed by the compact structure of the coacervates that might be originated from the Ostwald ripening (Singh et al., 2007). The OD_600_ reached the highest values severally around pH 3.2, 3.5, and 3.9 for the HSPI/SA ratio of 5:1, 7:1, and 9:1, respectively. As the pH further decreased, the OD_600_ value decreased from the peak value and the decline rate was slower than that in the ascending stage. At this time, the electrostatic interaction force gradually weakened.

Generally, different peak values were obtained with the different HSPI/SA ratio. The peak value obtained with the HSPI/SA ratio of 7:1 at the pH 3.5 was higher than that with another two mixture mass ratios, indicating a preferable complex coacervate. Therefore, the theoretical pH value for the HSPI/SA complex coalescence in this study is 3.5 and the HSPI/SA ratio is 7:1.

### 3.6 Analysis for FTIR

**FIGURE 6:**
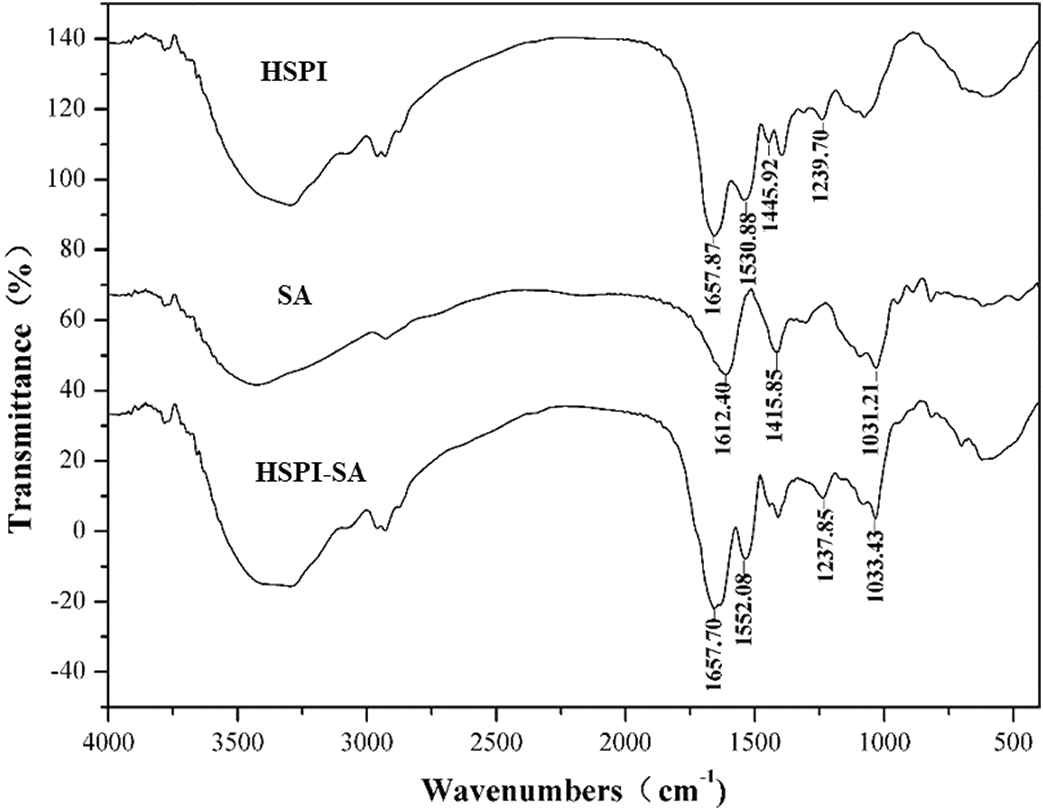
FTIR of HSPI, SA, and HSPI‐SA

Figure 6 illustrated the infrared spectrums of HSPI, SA, and HSPI/SA coacervate. Amides I, II, and III are the most sensitive regions in FTIR to the conformational changes of the protein secondary structure. As for HSPI, the characteristic peaks appeared at 1657.87, 1538.88, and 1239.70 cm^−1^, respectively. Among them, the peak at 1657.87 cm^−1^ was the amide I band, which could be attributed to the C = O stretch vibration. The amide II band at around 1530.88 cm^−1^ was assigned to the ‐N‐H flexural vibrations, and the amide III band at 1239.70 cm^−1^ was attributed to stretching vibration of the N‐H and C‐H groups. In the spectrum of SA, the strong absorption bands at around 1612.40 and 1415.85 cm^−1^ were assigned to the ‐COO^−^ asymmetric and symmetrical telescopic vibration, respectively. In addition, the absorption peak at a wavenumber of 1031.21 cm^−1^ represented the stretching vibration of C‐O (Muhoza et al., 2019).

Comparing HSPI with the HSPI/SA coacervation, there was a slight shift in the ‐N‐H flexural vibrations peaks from 1530.88 to 1552.08 cm^−1^ (Duhoranimana et al., 2017). In addition, comparing SA with the HSPI/SA coacervation, the peaks at 1612.40 and 1415.85 cm^−1^ for ‐COO^−^ stretching vibration also disappeared. Therefore, experimental results confirmed that the reaction between SA and HSPI was attributed to the electrostatic interaction between ‐NH_3_^+^ in HSPI and ‐COO^−^ in SA. It also demonstrated that during the formation of the complexes, only electrostatic interactions occurred.

### 3.7 Analysis for ITC

**FIGURE 7:**
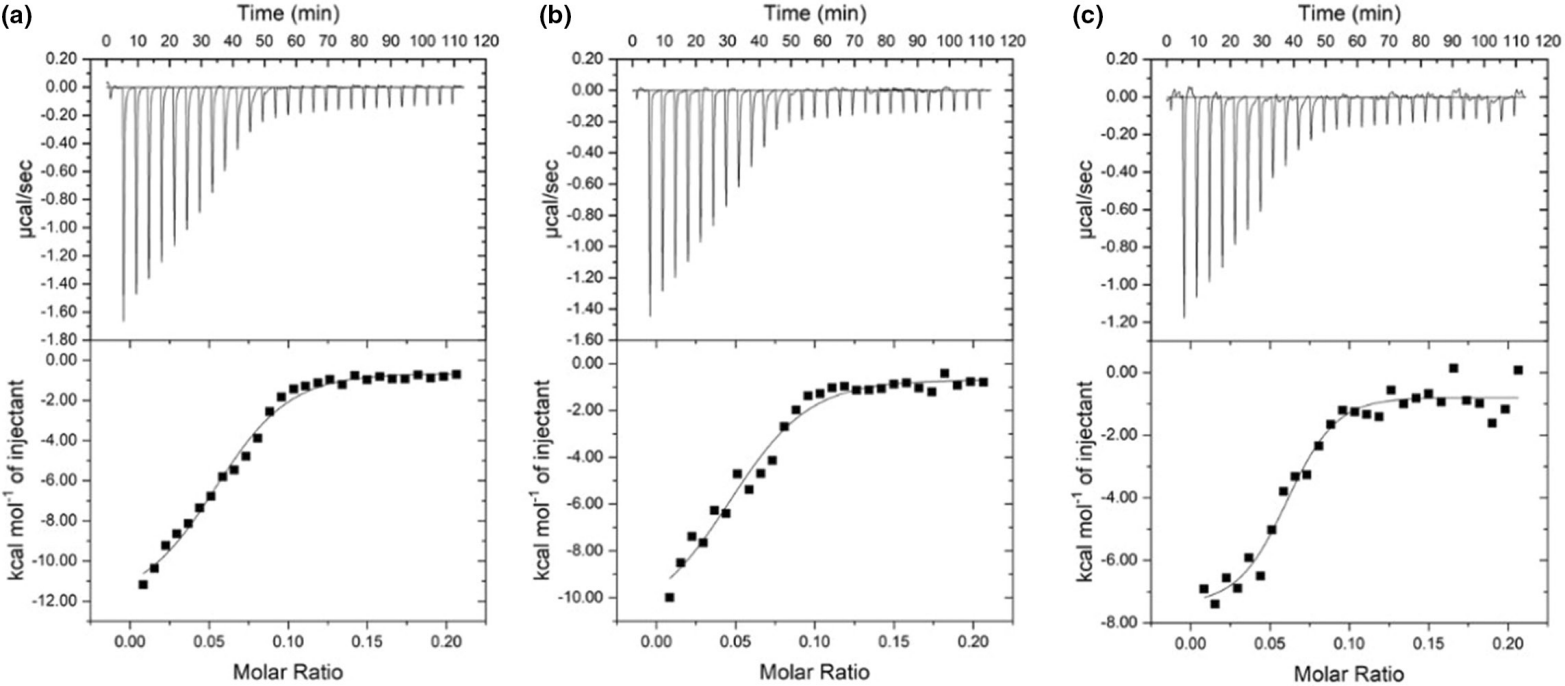
Isothermal titration calorimetry titration of SA with HSPI at (a) T1 = 25°C, (b) T2 = 35°C, and (c) 45°C

Isothermal titration calorimetry could be applied to reveal the interactions, binding affinity, and thermodynamics of complex coacervate reaction (Li et al., 2020). The advantage of ITC is that it sensitively measures enthalpy changes with regard to the interaction among molecules. Figure 7 presented the ITC thermograms of the heat flow as a function of time from the titration of HSPI with SA at 25, 35 and 45°C. The integral of titration peaks corresponds to the heat changes within titration. As shown in Figure 7, the height of the peaks first decreased and then became constant, which indicated the gradual saturation of HSPI binding sites. In addition, as shown in Figure 7c, there were outliers between the integrated enthalpy change values and the baseline in the titration curve at 45°C. This might be attributed that the increased reaction temperature led to the formation of larger cohesive groups in the complex reaction (Kaspchak et al., 2018).

The thermodynamic parameters such as affinity constant (K), enthalpy change (∆H), entropy change (∆S), Gibbs free energy (∆G), as well as interaction forces between two substances during reaction process could be calculated based on the ITC fitting model (Emaga et al., 2012). The titration thermodynamic parameters of SA and HSPI at different temperatures were shown in Table 1. According to the affinity index K, the values between at 25 and 45°C were higher than that at 35°C, which indicated that the HSPI/SA binding strength was the weakest at 35°C. Copreservation of complexes is thermodynamically favorable when ΔG is negative (Kayitmazer, 2017). The negative ΔH represents an exothermic change. The binding process was enthalpically driven with ΔH of −13, −12.6 and −8.8 kcal/mol at 25, 35 and 45°C, respectively. As the absolute values of ΔH were in descending order severally at the temperature of 25, 35 and 45°C, the formation of HSPI/SA complex is more favorable at 25°C than that at 35 and 45°C. Furthermore, the negative ΔH and ΔS were observed in the complexes of HSPI and SA, which suggested that the binding interaction forces were governed by the noncovalent nature and the interaction is spontaneous (Hadian et al., 2016; Zhan et al., 2018).

**TABLE 1:** Thermodynamic parameters of binding between SA and HSPI at different temperatures

Temperature (°C)	*K* (M^−1^) × 10^5^	*∆H* (kcal/mol)	*T∆S* (kcal/mol)	*∆G* (kcal/mol)
25	1.83 ± 0.27	−13.0 ± 0.65	−5.8404	−7.2 ± 0.65
35	1.17 ± 0.28	−12.6 ± 1.34	−5.4203	−7.2 ± 1.34
45	2.01 ± 0.60	−8.8 ± 0.76	−1.0907	−7.7 ± 0.76

As the complex polymer HSPI/SA consist of two kinds of polyelectrolytes, the ITC results could be further explained by the titration endpoint. Since the relative molecular mass of HSPI was roughly equivalent to that of SA, the molar concentration ratio of HSPI/SA in this reaction was approximately equal to its mass concentration ratio. It could be found from the titration enthalpy curve at 25°C (Figure 7a) that the endpoint of the titration of HSPI with SA occurred at the molar ratio of 0.14, which is equivalent to the mass ratio of 7:1 for HSPI/SA. The ITC result was basically consistent with the result of turbidity titration.

### 3.8 Thermogravimetric analysis (TGA) of microcapsules

**FIGURE 8:**
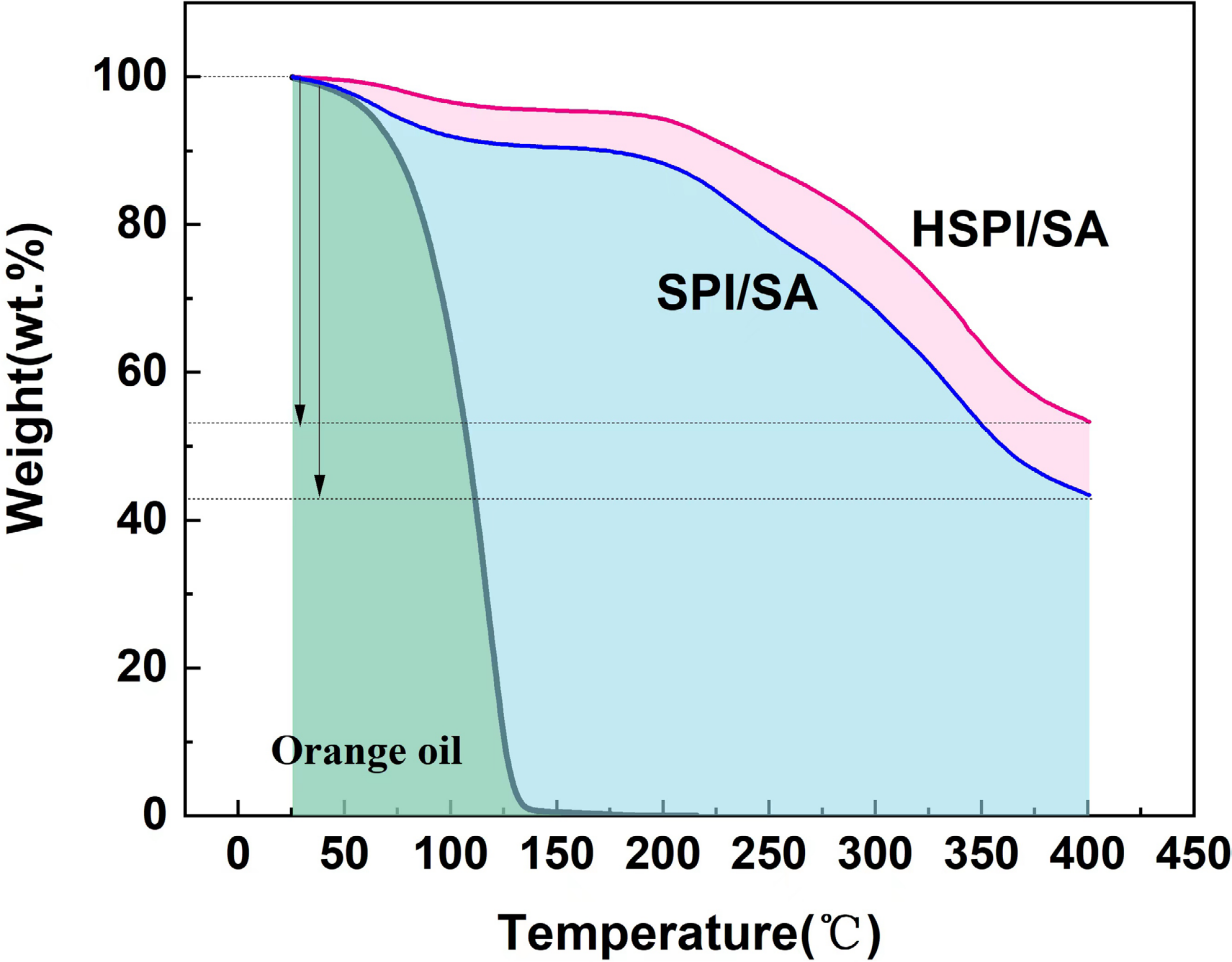
Thermogravimetric analysis of sweet orange oil and complex coalescing microcapsules

Thermogravimetric analysis was used to observe the effect of storage temperature on the weight loss curve, which was mainly determined by the evaporation of microcapsules (Haleva‐Toledo et al., 1999). The TGA curves of sweet orange oil, HSPI/SA, and SPI/SA from 0 to 400°C were shown in Figure 8. TGA curves of the unembedded sweet orange oil and two kinds of microcapsules had no obvious change at the initial stage. However, when the temperature was increased above 75°C, the mass loss of sweet orange oil was higher, and when the temperature increased to 150°C, the mass loss was close to 100%. The mass loss of the two microcapsules increased with the variation of temperature. The maximum mass loss of the two microcapsules occurred at the heating temperature of 200–350°C. At this time, the mass loss of HSPI microcapsules reached 37.2% and the ISC microcapsules reached 44.7%. As shown in Figure 8, during the heating process, the weight of HISC was always higher than that of ISC, indicating that the heat resistance of HSPI microcapsules was better than that of SPI microcapsules.

## 4 CONCLUSION

This study demonstrates that enzymatic digestion of soy protein isolate (SPI) with F400 at the DH of 2% could improve its heat stability, emulsibility, and inhibiting activity of lipid oxidation. Subsequently, HSPI and SA were polymerized and the formation mechanism of the composite coalescence reaction and its application as microencapsulation was investigated.

1. The optimal mass ratio of HSPI/SA in the preparation of complex polymer was 7:1 as provided by turbidity measurements. Meanwhile, the complex reaction of HSPI with SA could be attributed to the electrostatic interaction between ‐NH_3_^+^ in HSPI and ‐COO^−^ in SA based on the FTIR spectroscopic analysis. The results of the ITC study manifested that the enthalpy of the HSPI/SA complex formation phase was negative, indicating an enthalpy‐driven reaction in the composite process.
2. HSPI/SA composite system possessed higher heat resistance and affinity to the core material compared with SPI/SA according to the TGA analysis. Therefore, HSPI/SA was found more suitable for being applied as wall material of microencapsulation.

## CONFLICT OF INTEREST

The authors declare no conflict of interest.

## Data Availability

The data for thermodynamic analysis of SA and HSPI at different temperatures are in the public domain as described in Table 1.
